# A Meta-Analysis of the Rates of Suicide Ideation, Attempts and Deaths in People with Epilepsy

**DOI:** 10.3390/ijerph16081451

**Published:** 2019-04-24

**Authors:** Nicholas Abraham, P. Buvanaswari, Rahul Rathakrishnan, Bach X. Tran, Giang Vu. Thu, Long H. Nguyen, Cyrus S. Ho, Roger C. Ho

**Affiliations:** 1 Department of Psychological Medicine, Yong Loo Lin School of Medicine, National University of Singapore, Singapore 119228, Singapore; nicholasjeyabraham@gmail.com (N.A.); pcmrhcm@nus.edu.sg (R.C.H.); 2 Department of Psychological Medicine, National University Health System, Singapore 119228, Singapore; su_hui_ho@nuhs.edu.sg; 3 Division of Neurology, University Medical Cluster, National University Health System, Singapore 119228, Singapore; rahul_rathakrishnan@nuhs.edu.sg; 4 Institute for Preventive Medicine and Public Health, Hanoi Medical University, Hanoi 100000, Vietnam; bach.ipmph@gmail.com; 5 Johns Hopkins Bloomberg School of Public Health, Johns Hopkins University, Baltimore, MD 21205, USA; 6 Vietnam Young Physicians’ Association, Hanoi 100000, Vietnam; 7 Center of Excellence in Evidence-based Medicine, Nguyen Tat Thanh University, Ho Chi Minh City 70000, Vietnam; gigi.vugiang@gmail.com; 8 Center of Excellence in Behavioral Medicine, Nguyen Tat Thanh University, Ho Chi Minh City 70000, Vietnam; longnh.ph@gmail.com; 9 Biomedical Global Institute of Healthcare Research & Technology (BIGHEART), National University of Singapore, Singapore 119228, Singapore

**Keywords:** epilepsy, meta-analysis, prevalence, suicide

## Abstract

This meta-analysis aimed to evaluate the association between epilepsy and suicide. We systematically searched PubMed, PsycINFO, Embase and Web of Science for studies that reported the prevalence of suicidality in the form of suicide ideation, attempts and deaths among people with epilepsy (PWE). Studies were included if they reported the numbers of patients who died by suicide and concurrently suffered from epilepsy, assessed suicide ideation, or studied suicide attempts in PWE by validated instruments or diagnostic interviews. We used the random effects model to calculate the pooled odds ratios (OR) and standard mean differences (SMDs). We performed subgroup analyses. Seven case-control studies were included in the comparison of rates of suicide attempts between PWE and controls, with a total of 821,594 participants. Our analyses demonstrated a positive association between epilepsy and suicide attempts (pooled OR = 3.25, 95% confidence interval (CI): 2.69–3.92, *p* < 0.001), indicating that PWE have an elevated risk of suicide. The pooled prevalence for suicide ideation (24 studies) and suicide attempts (18 studies) were 23.2% (95% CI: 0.176–0.301) and 7.4% (95% CI: 0.031–0.169) respectively. The pooled rate of death due to suicide (10 studies) was 0.5% (95% CI: 0.002–0.016). Meta-regression showed that mean age and proportion of male gender were significant moderators for prevalence of suicide attempts and death due to suicide in PWE. Young PWE could be triggered by relationship problems and male PWE might use more lethal methods to attempt suicide. This meta-analysis provides the most up-to-date information on the prevalence of suicide among people with epilepsy and guidance on strategies to improve current psychiatric services provided for this population.

## 1. Introduction

Epilepsy is a significant disease burden among the worldwide population. A recent meta-analysis found the median incidence of epilepsy per 100,000 people per year to be 45.0 in high income economies and 81.7 per 100,000 people in the rest of the world [1]. Suicide is associated with symptoms of psychiatric illness [2], psychosomatic complaints of insomnia and headaches [3], reports of adverse life events [4], alcohol abuse and past medical or surgical treatment [5], but its association with epilepsy, globally, has not been quantified. The reported percentages of suicidality among people with epilepsy (PWE) vary amongst studies. Additionally, the clinical heterogeneity of epilepsy does not allow a proper comparison between different samples of patients who might have different subtypes of epilepsy or have had differing pharmacological or surgical interventions. The relationship between epilepsy and suicide is complex. The US Food and Drug Administration (FDA) concluded that antiepileptic drugs increase the risk for suicidal thoughts [6]. In contrast, Hesdorffer et al. found that suicide attempts were associated with incident epilepsy in the absence of antiepileptic drugs in American patients [7]. People with new onset of epilepsy are vulnerable to suicide [8]. Jones et al.. reported that there was overestimation of suicide in PWE who attended tertiary outpatient clinics, but suicidal behaviour was found to be common in PWE who sought treatment in the community settings [9]. Meyer et al. found that PWE who self-harmed (inclusive of suicide attempts) did so more frequently than other self-harm patients [10]. Previous meta-analyses only reported the number of deaths by suicide in PWE but not the prevalence of suicide ideation and attempts [11,12]. We conducted a meta-analysis to determine the association between any form of epilepsy and suicidal ideation, suicide attempts, as well as death due to suicide.

## 2. Materials and Methods

### 2.1. Search Strategy

We conducted a systematic search of PubMed, PsycINFO, Embase and Web of Science from inception to 22 April 2018. The key terms used in the search were referenced epilepsy (epilep*, epilepsy, epileptic), seizures (seiz*, seizure, seizures, seizing), and suicidality (suicide*, suicidality, suicide, suicides, suicidal). We did not include the term “deliberate self-harm” (DSH) as a search term, as when only DSH is reported without reference to suicidality, it is not possible to differentiate suicidal from non-suicidal self-harm [13]. Using these search parameters, we identified 1301 studies for review. Additionally, the reference lists of all studies of interest were manually examined and a total of 53 studies were identified for further evaluation.

### 2.2. Inclusion and Exclusion Criteria

We included the studies that examined the association between epilepsy and suicide in all patients. We only included studies that had assessed suicidality based on diagnostic interviews (for example, but not limited to, Mini International Neuropsychiatric Interview (MINI-Plus)) or other questionnaires and diagnostic tools (for example, but not limited to, Neurological Disorders Depression Inventory for Epilepsy (NDDI-E), Hamilton Depression Rating Scale (HDRS), and Beck Depression Inventory (BDI)). For the diagnosis of epilepsy, we also only included studies that had quantified the number of PWE based on a diagnosis established by clinicians or a diagnosis that had been reflected in the patient’s medical records.

### 2.3. Statistical Analysis

We used the meta-analysis software Comprehensive Meta-analysis (CMA, BioStat Solutions, Inc, Frederick, MD, USA, version 3.0) to calculate the effect sizes and calculate the pooled mean effect size with the random effects model. The random effects model was used, as it assumes varying effect sizes between studies, because of differing study design and study population [14]. We converted the published data into odds ratios (OR). 

The studies were classified and analysed based on the number of PWE who had one of the following three outcomes: suicidal ideation, suicide attempts, and death from suicide. For case-control studies, the ORs for the number of PWE who attempted suicide were calculated with reference to the number of controls without epilepsy who attempted suicide. A Forest plot was then constructed for comparison, for which we reported the pooled OR, 95% confidence interval (CI) and p-value based on the method used by a previous meta-analysis [15]. The statistical significance level was set at *p* < 0.05. For the studies that reported outcomes, including number of patients with suicidal ideation, suicide attempts, and deaths attributed to suicide without a control group, pooled prevalence rates were calculated. To assess for publication bias, we conducted Egger’s test [16] and fail safe-N tests [17]. The $I^{2}$ statistic was used to assess the between-study heterogeneity [18], which described the percentage of variance on the basis of real differences in study effects. An $I^{2}$ value of 25% was considered low heterogeneity, 50% moderate and 75% substantial [19].

## 3. Results

**Figure 1:**
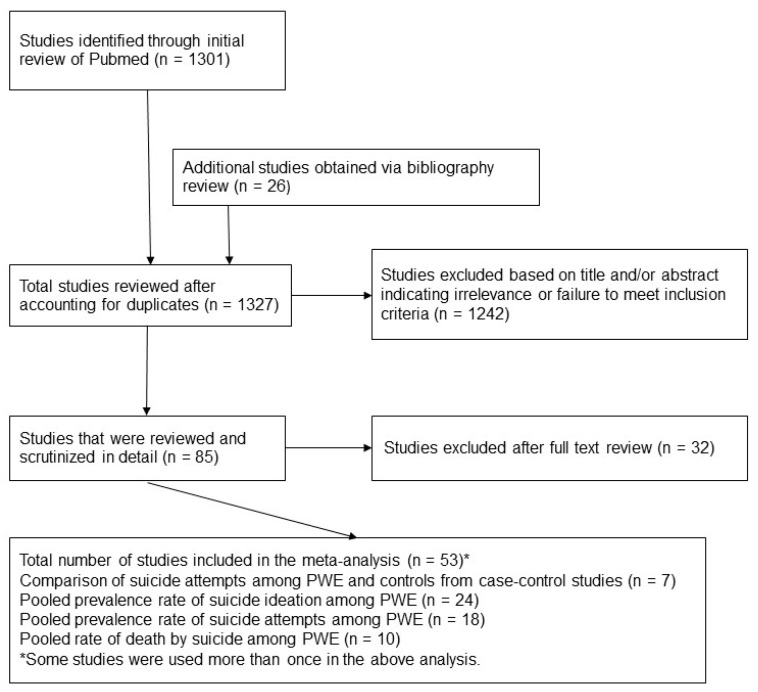
Process of systematic selection of articles. PWE, people with epilepsy.

The process of study selection is shown in Figure 1. A total of 7 case-control studies were included with 107,112 PWE, 714,482 controls without epilepsy for comparison and a total sample size of 821,594. Characteristics of the study population are presented in Table 1. 

**Figure 2:**
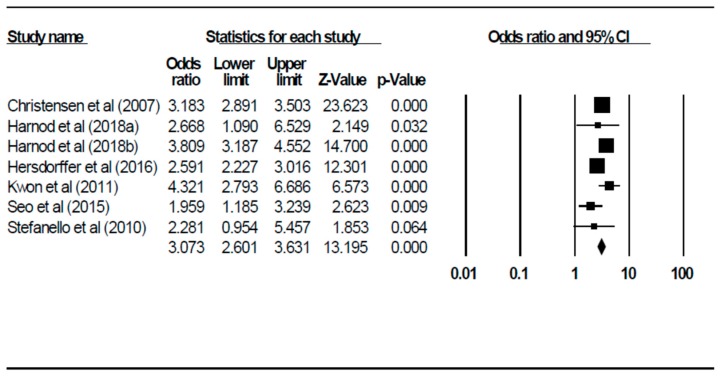
Forest plot of pooled odds ratio of suicide attempts among PWE and controls from case-control studies. CI, confidence interval.

Figure 2 shows the results of the 7 case-control studies that measured the association between suicide attempts and epilepsy. The pooled OR was 3.07 (95% confidence interval (CI): 2.60–3.63, *p* < 0.001) using the random effects model. This finding suggests that PWE were 3.07 times more likely to have made a suicide attempt than individuals who did not suffer from epilepsy. The $I^{2}$ value was 64.22 and it was statistically significant (*p* = 0.001). The mean age of PWE (B = 0.015, 95% CI: 0.005–0.026, z = 2.857, *p* = 0.004) was identified as a significant moderator that contributed to heterogeneity between the studies. The percentage of male patients (β = 0.89, 95% CI: −0.14–1.92, z = 1.70, *p* = 0.09) was a non-significant moderator. There was no evidence of publication bias (intercept = 0.74, 95% CI: −1.31–3.51, t = 0.75, df = 5, *p* = 0.43).

**Figure 3:**
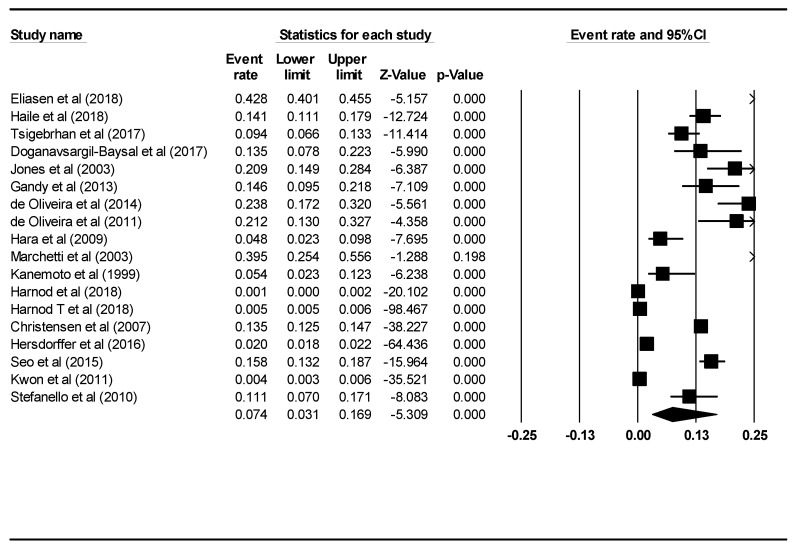
Forest plot of pooled prevalence rate of suicide attempts among PWE.

We also analysed the number of PWE who had attempted suicide among PWE in studies that did not include a control group. A total of 18 studies, with a total sample size of 109,938 PWE, were analysed. Figure 3 demonstrates the pooled prevalence rate of suicide attempts among PWE of these 18 studies. The pooled prevalence rate for suicide attempts was 0.074 or 7.4% (95% CI: 0.031–0.169, *p* < 0.001) using the random effects model. This suggests that 7.4% of PWE had a history of suicide attempts. The $I^{2}$ value was 99.7, which was statistically significant (*p* < 0.001). The percentage of males (B = 3.89, 95% CI: 3.18–4.61, z = 10.67, *p* < 0.001) and mean age (B = −0.12, 95% CI: −0.12–−0.11, z = −36.77, *p* < 0.001) were significant moderators that contributed to heterogeneity between the studies. There was no evidence of publication bias (intercept = 1.58, 95% CI: −13.39–16.57, t = 0.22, df = 16, *p* = 0.83).

**Figure 4:**
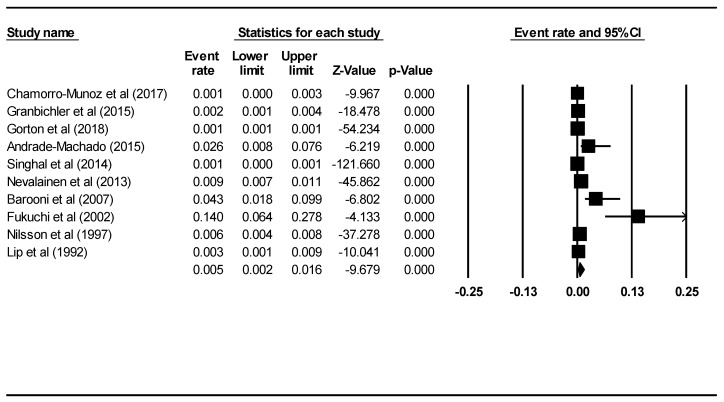
Forest plot of pooled rate of death by suicide among PWE.

A total of 10 studies, with a total sample size of 595,605, were analysed for a pooled prevalence rate of death due to suicide among PWE. Figure 4 demonstrates the results of the 10 studies that measured the pooled death rate due to suicide among PWE. The pooled death rate due to suicide was 0.005 or 0.5% (95% CI: 0.002–0.016, *p* < 0.001) using the random effects model. After removing an outlier study by Fukuchi et al. [25] the pooled death rate due to suicide was 0.004 or 0.4% (95% CI: 0.001–0.011, *p* < 0.001) using the random effects model. The $I^{2}$ value was 99.02, which was statistically significant (*p* < 0.001). The percentage of males (B = 25.28, 95% CI: 22.94–27.62, z = 21.24, *p* < 0.001) and mean age (B = −0.04, 95% CI: −0.07–−0.01, z = −2.47, *p* = 0.01) were significant moderators that contributed to heterogeneity between the studies. There was no evidence of publication bias (intercept = 7.61, 95% CI: −2.51–17.72, t = 1.73, df = 8, *p* = 0.12).

**Figure 5:**
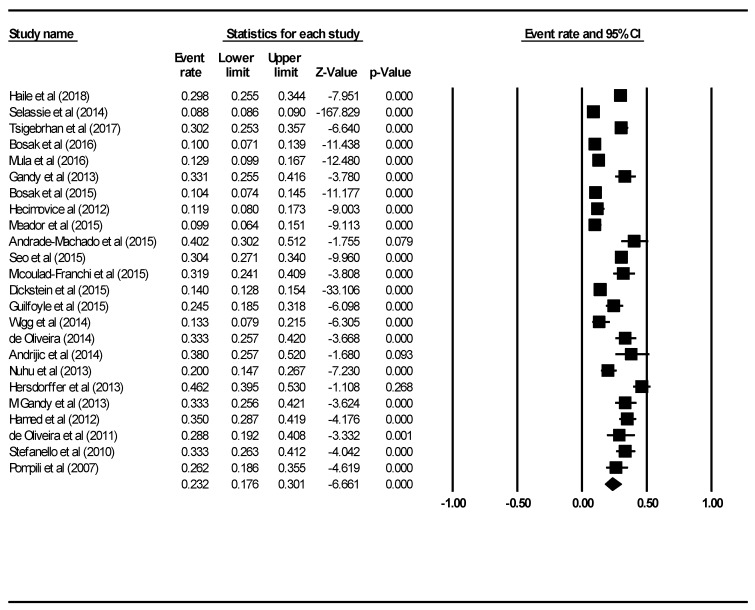
Forest plot of pooled rate of rate of suicide ideation among PWE.

We also analysed studies to quantify the rate of suicide ideation among PWE. A total of 24 studies, with a total sample size of 71,328 were analysed. Figure 5 demonstrates results of the 24 studies that measured the association between suicide ideation and epilepsy. The pooled event rate was 0.232 or 23.2% (95% CI: 0.176–0.301, *p* < 0.001) using the random effects model. The $I^{2}$ value was 98.43, which was statistically significant (*p* < 0.001). The percentage of males (B = 5.24, 95% CI: 4.61–5.86, z = 16.39, *p* < 0.001) and mean age (B = −0.05, 95% CI: −0.06–−0.04, z = −13.56, *p* < 0.01) were significant moderators that contributed to heterogeneity between the studies. There was evidence of publication bias (intercept = 7.35, 95% CI: 4.85–9.85, t = 6.08, df = 23, *p* < 0.01). 

## 4. Discussion

This meta-analysis was conducted to quantify the associations between epilepsy and suicidality, globally, in the forms of suicide ideation, suicide attempts, and death due to suicide. In the meta-analysis of seven studies with 821,954 participants in total, we found a significant association between suicide attempts and epilepsy (OR = 3.25; 95% CI: 2.69–3.92, *p* < 0.001). Our results corroborate previous studies, which concluded that a clear relationship between epilepsy and suicidality exists [11,26]. The results of this meta-analysis are also similar to those quantifying the rate of suicide attempts amongst people with other neurological diseases [12], indicating that suicidality is increased among people with epilepsy and other neurological disorders.

The World Health Organization (WHO) approximated that about 800,000 people die from suicide per year [27]. In this meta-analysis, the pooled event rate of completed suicide among PWE was 0.5%, which is higher than the rate of completed suicide (0.01%) among the general population as quantified by the WHO [27]. This suggests that the risk of completed suicide among PWE is higher than in the general population.

Among the 23 studies that measured the association between suicidal ideation and epilepsy, we analysed that the pooled event rate was 23.2%. In contrast, the prevalence of suicidal ideation in the general population was reported to be 3.1% in a cross-national study including 17 countries [28]. The prevalence of suicidal ideation reported in patients suffering from neurological diseases including stroke and epilepsy ranges from 5.7 to 12.7% [29,30,31]. In this meta-analysis, meta-regression showed that age was a significant moderator for pooled odds ratio and prevalence of suicide attempts in PWE. Previous studies found that factors precipitating suicide attempts varied across the lifespan, with suicide attempts by younger adults being triggered more by relationship problems and those by older adults being triggered more by financial and/or medical problems [32]. In the studies on PWE, the proportion of male gender was a significant moderator for the pooled prevalence of suicide ideation and death rate due to suicide. The regression coefficient for proportion of male gender is positive, which means that increase in proportion of male gender corresponds to an increase in effect size (i.e., the prevalence of suicide ideation, attempts or death by suicide in PWE). It has been reported that men tend to attempt suicide using methods perceived to be more lethal than the methods used by women [33], which could account for this effect.

### 4.1. Areas for Future Research

Overall, we found that there were few studies that compared the prevalence rate of suicidal ideation or death due to suicide between PWE and healthy controls. Having a control group would allow better comparison and better understanding of the increased risk of suicidality among PWE. Further research is required to determine the rate of PWE seeking psychiatric or pharmacological intervention for suicidality and other psychiatric co-morbidities, as well as the factors which influence this. This research will identify the treatment gap that prevents this potentially vulnerable group from seeking psychiatric help and guide the improvement of psychiatric services provided for people with epilepsy. Furthermore, the prevalence rate of suicidal ideation or death by suicide should be compared with a control group that has other challenging conditions including patients with psychiatric conditions.

### 4.2. Limitations

This study has several inherent limitations, which are commonly found in meta-analyses of prevalence studies. Firstly, the included studies showed a high level of heterogeneity. Heterogeneity appears to be the norm rather than the exception in meta-analyses of large numbers of cross-sectional studies globally [34]. Secondly, due to limited data, a meta-regression could not be performed on important moderators, such as factors predicting medical lethality [32], ethnicity [35], suicide method [2], substance abuse [3], history of suicide attempts [3] and other medical comorbidities [36]. Thirdly, meta-regression represents an observational association and is limited by ecological fallacy [37], and cross-sectional information cannot establish causality. More longitudinal studies are required to determine the temporal association between predisposing factors and the risk of developing suicidality. Fourthly, we conducted our search only for articles in English, and thus may have omitted relevant studies if they were not available in English. Lastly, the original studies included in this meta-analysis did not specify the type of epilepsy. As a result, this meta-analysis could not include subgroup analysis to compare the prevalence rate of suicide ideation, attempts and death by suicide in different types of epilepsy. Previous studies found that individuals with temporal lobe epilepsy were noted to have a 6–25 times increased risk of suicidal ideation [38], while those with complex partial seizures had up to a 25 times increased risk of suicide [39]. Despite these limitations, this meta-analysis provides the most up-to-date information on the prevalence of suicide among people with epilepsy. 

## 5. Conclusions

Our meta-analysis has indicated that suicidality in people with epilepsy (PWE) is more common than among the general population in the context of suicide attempts. Additional analysis has also shown that other indicators of suicide risk, including suicidal ideation and death by suicide, are also increased among PWE. This highlights a need for improved suicide risk screening, as well as the identification and implementation of preventive and precautionary strategies, for people with epilepsy. 

## Figures and Tables

**Table 1:** Descriptive data of the seven included case-control studies.

Author	Year of Publication	Country	Source of Data	Number of PWE Who Attempted Suicide	Total Number of PWE	Number of Controls Who Attempted Suicide	Total Number of Controls	Odds Ratio	Mean Age (PWE)	Mean Age (Controls)	Male % (PWE)	Male % (Controls)	Score Based on the Newcastle-Ottawa Scale
Christensen et al. [8]	2007	Denmark	National registry	492	3632	20,677	440,665	3.18	Not available	Not available	68.4	64.6	9
Harnod et al. [20]	2018	Taiwan	General hospital	8	9801	12	39,204	2.67	9.9	9.89	55.7	55.7	9
Harnod et al. [21]	2018	Taiwan	National Health insurance database	351	68,543	185	137,086	3.81	56.4	55.9	62.9	62.9	9
Hersdorffer et al. [7]	2016	United Kingdom	General practice	278	14,059	434	56,184	2.59	36	36	51.8	51.8	6
Kwon et al. [22]	2011	Canada	Administrative database	42	10,240	39	40,960	4.32	39	39	51.5	51.5	9
Seo et al. [23]	2015	Koreas	Secondary and tertiary clinics	108	684	20	229	1.96	41.5	41.4	57.6	57.2	8
Stefanello et al. [24]	2010	Brazil	City and rural areas	17	153	8	154	2.28	Not available	Not available	54.2	55.2	7
